# A novel m7G-related miRNA prognostic signature for predicting clinical outcome and immune microenvironment in colon cancer

**DOI:** 10.7150/jca.99173

**Published:** 2024-10-07

**Authors:** Zhenghui Zhu, Yuxia Xie, Minhao Yin, Lei Peng, Hong Zhu

**Affiliations:** Department of Gastroenterology, The First Affiliated Hospital of Nanjing Medical University, Nanjing, Jiangsu, China.

**Keywords:** colon cancer, N7-methylguanosine, microRNA, prognostic signature, immune microenvironment

## Abstract

**Background:** Colon cancer (CC) is a highly prevalent malignancy worldwide, characterized by elevated mortality rates and poor prognosis. N7-methylguanosine (m7G) methylation is an emerging RNA modification type and involved in the development of many tumors. Despite this, the correlation between m7G-related miRNAs and CC remains to be elucidated. This research aimed to investigate the clinical significance of m7G-related miRNAs in predicting both the prognosis and tumor microenvironment (TME) of CC.

**Method:** We retrieved transcriptome data and associated clinical information from a publicly accessible database. Using univariate Cox and LASSO regression analyses, we established a signature of m7G-related miRNAs. Additionally, we used CIBERSORT and ssGSEA algorithms to explore the association between the prognostic risk score and the TME in CC patients. By considering the risk signature and immune infiltration, we identified differentially expressed genes that contribute to the prognosis of CC. Finally, the expression patterns of prognostic miRNAs were verified using quantitative reverse transcriptase PCR (qRT-PCR) in cell lines.

**Results:** We constructed a prognostic risk signature based on seven m7G-related miRNAs (miR-136-5p, miR-6887-3p, miR-195-5p, miR-149-3p, miR-4433a-5p, miR-31-5p, and miR-129-2-3p). Subsequently, we observed remarkable differences in patient outcomes between the high- and low-risk groups. The area under the curve (AUC) for 1-, 3-, and 5-year survivals in the ROC curve were 0.735, 0.707, and 0.632, respectively. Furthermore, our results showed that the risk score can serve as an independent prognostic biomarker for overall survival prediction. In terms of immune analysis, the results revealed a significant association between the risk signature and immune infiltration, as well as immune checkpoint expression. Finally, our study showed that CCDC160 and RLN3 is the gene most relevant to immune cells and function in CC.

**Conclusion:** Our study conducted a comprehensive and systematic analysis of m7G-associated miRNAs to construct prognostic profiles of CC. We developed a prognostic risk model based on m7G-miRNAs, with the resulting risk scores demonstrating considerable potential as prognostic biomarkers. These findings provide substantial evidence for the critical role of m7G-related miRNAs in colon cancer and may offer new immunotherapeutic targets for patients with this disease.

## Introduction

Colorectal cancer (CRC) holds the third position in terms of its incidence among diagnosed cancers and is the second most common cause of cancer-related deaths globally^1^. In recent years, there has been a notable increase in the incidence of colon cancer (CC), accompanied by a decreased age at diagnosis among colorectal cancer patients^2^. Early-onset colorectal cancer (EoCRC), defined as newly diagnosed cases occurring in adults under the age of 50, accounts for around 10% of all cases^3^. Currently, surgery, chemotherapy, radiotherapy, and biotherapy are used to treat CC. Despite some advances in diagnostic methods and therapeutic approaches, the clinical outcomes of CC patients remain poor due to advanced stage, aggressive progression, and early metastasis^4^. A significant proportion of colon cancer (CC) patients, approximately 40%, unfortunately encounter tumor relapse or late metastasis, with a survival rate of less than 15% after 5 years^5^. Therefore, there is a critical need to identify novel diagnostic and therapeutic biomarkers that can accurately assess the prognosis of CC patients.

RNA methylation is a widespread post-transcriptional modification that exists in both eukaryotes and prokaryotes. Extensive evidence has demonstrated its association with a wide range of biological processes and diseases^6^, ^7^. RNA methylation can occur at different sites, including N6-methyladenosine (m6A), N5-methylcytosine (m5C), N7-methylguanosine (m7G) and 2-O-methylation modifications^8^. Among them, the m7G modification occurs not only at the 5' cap of mRNA but also internally in mRNA, rRNA, tRNA, and even microRNA (miRNA) 9, 10, 11, 12, 13. Recent research has highlighted the vital role of the m7G methylation in regulating tumor development related biological processes. METTL1 and WDR4 are the best-characterized m7G regulators in human beings^14^. Multiple studies have demonstrated the involvement of m7G regulators in tumor progression through their regulation of crucial factors such as tumor immunity, metabolic reprogramming, and drug resistance^15^. For instance, let-7e miRNA exhibits mutual interactions with METTL1 by mediating m7G methylation, which inhibits the downstream target gene HMGA2 and suppresses the progression of CC. Overall, these findings demonstrate the complex and multifaceted nature of RNA methylation and its importance in disease pathology, particularly in cancer.

MicroRNAs (miRNAs) are small, conserved non-coding RNAs that play a crucial role in post-transcriptional gene regulation by binding to target mRNAs^15^. Extensive research has provided compelling evidence of the involvement of miRNAs in a diverse array of cellular biological processes and pathogenesis of various diseases, with particular emphasis on cancer^16^, ^17^. miRNAs exert a substantial influence on the progression and metastasis of CC by regulating key signaling pathways, including Wnt/β-catenin, PI3K/AKT/mTOR, and ERK-MAPK^18^, ^19^. These findings suggest that miRNAs have the potential to serve as biomarkers for diagnosing, prognosticating, and targeting therapeutic interventions in CC^20^. The m7G modification represents a novel regulatory mechanism for miRNAs. Studies have shown that m7G in miRNA is involved in the regulation of its downstream target genes, thereby regulating the tumorigenesis and development. For example, m7G methylation promotes the process of pri-miRNA transcript into pre-miRNA and accelerates the maturation efficiency of miRNA, thus decreasing the expression of targeted genes HMGA2 and inhibiting the progression of colon and lung cancers^13^. Another study suggested that S100A4/p53 axis was the downstream target of METTL1 and miR-149-3p, and METTL1-mediated m7G methylation in miR-149-3p increased p53 protein levels in CC cells, which were reversed by upregulating S100A4^21^. Similarly, Xie et.al concluded that m7G modified sites in miR-760 at G-rich regions modulated by METTL1 methylation to accelerate the degradation of ATF3, which made a progression of proliferation and migration in breast cancer^22^. The above results revealed that m7G miRNA methylation selectively promotes the processing of certain miRNAs translation to regulate the expression of its downstream tumor-related genes, thereby promoting or inhibiting tumor progression. Nevertheless, the precise impact of m7G-related miRNAs on the prognosis of CC remains uncertain. Thus, further investigation is warranted to explore the potential of m7G-related miRNAs as biomarkers for CC patients.

## Materials and Methods

### Data extraction

The clinical information and sequencing data, including mRNA (483 cases of CC and 41 cases of normal tissue) and miRNA (457 cases of CC and 8 cases of normal tissue) sequencing expression profiles of CC were downloaded from The Cancer Genome Atlas (TCGA) database. A dataset comprising 442 complete CC samples with miRNA sequencing data and corresponding clinical information were obtained for further analysis after removing incomplete samples. To investigate the role of m7G modification on miRNA, METTL1 and WDR4 were selected based on previously research studies reporting their involvement in m7G modification.

### Identification of m7G-related differentially expressed miRNAs

Based on the human miRNA target gene file in TargetScan database, we obtained the m7G-related miRNAs. Subsequently, we extracted the expression matrix of these m7G-related miRNAs and performed differential analysis between tumor and normal samples using the “edge R” package. Specifically, we identified m7G-related DE-miRNAs (differentially expressed miRNAs) with FDR < 0.05 and |log FC| ≥ 1.

### Establishment and validation of the m7G-related miRNA model

We randomly divided the 442 CC samples into a training set and a testing set at a ratio of 1:1. The prognostic m7G-related miRNA model was established using the training set, while the testing set was used for model evaluation. Univariate Cox regression analysis was conducted on m7G-related DE-miRNAs to identify factors associated with prognosis (p < 0.05). The most significant prognostic m7G-related miRNAs were selected using the least absolute shrinkage and selection operator (LASSO) regression model. A prognostic signature that included 7 prognostic m7G-related miRNAs was obtained based on these results. We derived the individual risk score for each patient with colon cancer (CC) using the following formula: riskScore = 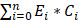, where E(i) represents the expression level of each miRNA in this model, and C(i) refers to the miRNA coefficient. We categorized the CC patients into high-risk and low-risk groups using the median risk score as the threshold. The “Rtsne” package was used to perform principal component analysis (PCA) of m7G-related DE-miRNAs based on the risk model. We utilized Kaplan-Meier survival analysis to evaluate the differences in overall survival (OS) among the different groups. To evaluate the accuracy and efficacy of the risk model, we utilized risk plots, receiver operating characteristic (ROC) curve analysis, and calculated the area under the curve (AUC) value.

### Construction of a nomogram based on riskScore and clinical predictors

We employed both univariate and multivariate Cox regression analyses to identify prognostic factors that are independent in CC patients. Subsequently, we developed a novel nomogram using the "rms" package, incorporating riskScore and other relevant clinicopathological characteristics. The prediction capacity of the miRNA-clinical nomogram was evaluated by computing the C-index and plotting calibration curves.

### Correlation between the risk model with immune filtration in CC

In this study, we employed the ESTIMATE algorithm to assess the disparities in TME scores and tumor purity between two groups. In addition, 29 immune-related gene sets were obtained using the "GSEABase" package for single sample gene set enrichment analysis (ssGSEA) to compare immune features among different risk score groups. Moreover, the stem cell properties of tumor were measured using mRNAsi, and the correlation between mRNAsi and two risk subgroups were examined. We employed Gene set enrichment analysis (GSEA) to gain insight into the biological processes associated with different subgroups. The hallmark gene set (h.all.v7.5.symbols.gmt), sourced from the MSigDB website, was utilized for this purpose.

### Analysis of differentially expressed mRNAs related with immunity and riskScore

To analyze the differentially expressed mRNAs (DE-mRNAs) between high-risk and low-risk samples, we employed the "edgeR" package. Furthermore, we classified patients into high- and low-immunity groups based on their immune scores. Risk-immunity-related mRNAs were obtained by intersecting risk differential mRNAs with immunity differential mRNAs (FDR < 0.05, |log FC | ≥ 1). To unearth the signaling pathways and biological processes in which these risk-immunity-related mRNAs were enriched, a GO analysis was conducted, with significant set at p < 0.05. Furthermore, we performed univariate Cox regression analysis of these risk-immunity-related mRNAs to screen for genes associated with prognosis (p < 0.05). Lastly, using Pearson correlation analysis, we explored the association between these prognosis-related genes and immune infiltration.

### qRT-PCR analysis

Total RNA was isolated from cells using the Trizol (RNAiso plus Takara Japan) Extraction Reagent. Later on, total RNA (1000ng) was reversely transcribed using miRNA 1st Strand cDNA Synthesis Kit (by tailing A) (Vazyme Biotechnology). After that, real-time PCR was run with StepOne™ Real-Time PCR System and Taq Pro Universal SYBR qPCR Master Mix (Vazyme Biotechnology). U6 was served as miRNA internal reference genes of miR-136-5p, miR-6887-3p, miR-195-5p, and miR-31-5p. Fold changes were calculated by using the 2-∆∆CT formula. The primers were purchased from Beijing Tsingke Biotech Co., Ltd. The primer sequences were as follows: U6, 5'-CTCGCTTCGGCAGCACAT-3'(F); miR-31-5p, 5'-GCGAGGCAAGATGCTGGC-3'(F); miR-136-5p, 5'-CGCGACTCCATTTGTTTTGAT-3'(F); miR-195-5p, 5'-GCGCGTAGCAGCACAGAAAT-3' (F); miR-6887-3p, 5'-GCGTCCCCTCCACTTTCC-3' (F). All reverse universal primers come from the miRNA 1st Strand cDNA Synthesis Kit's universal reverse Q primer.

## Results

### Identification of differentially expressed m7G-related miRNAs in CC

**Figure 1:**
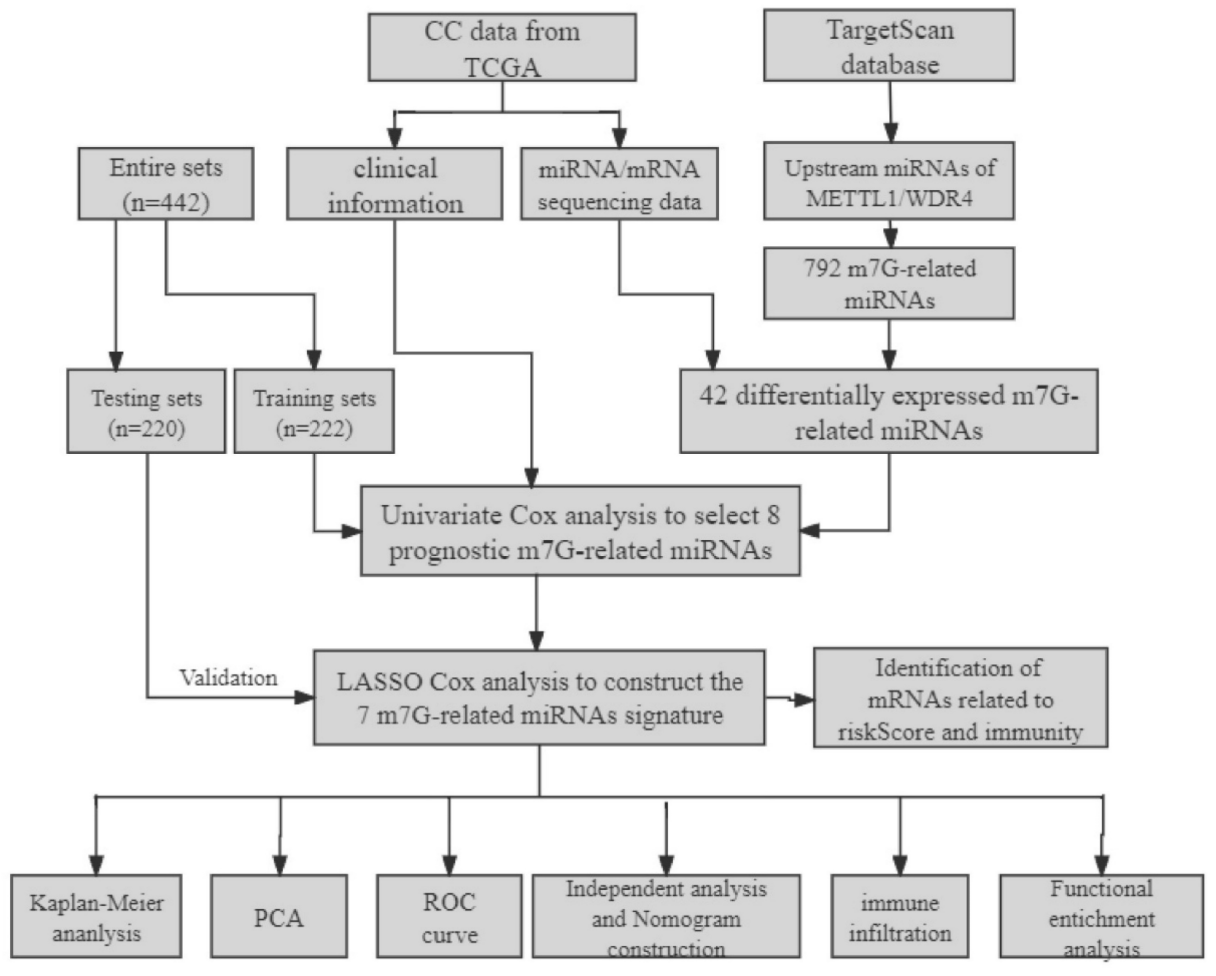
The flowchart of the study.

**Figure 2:**
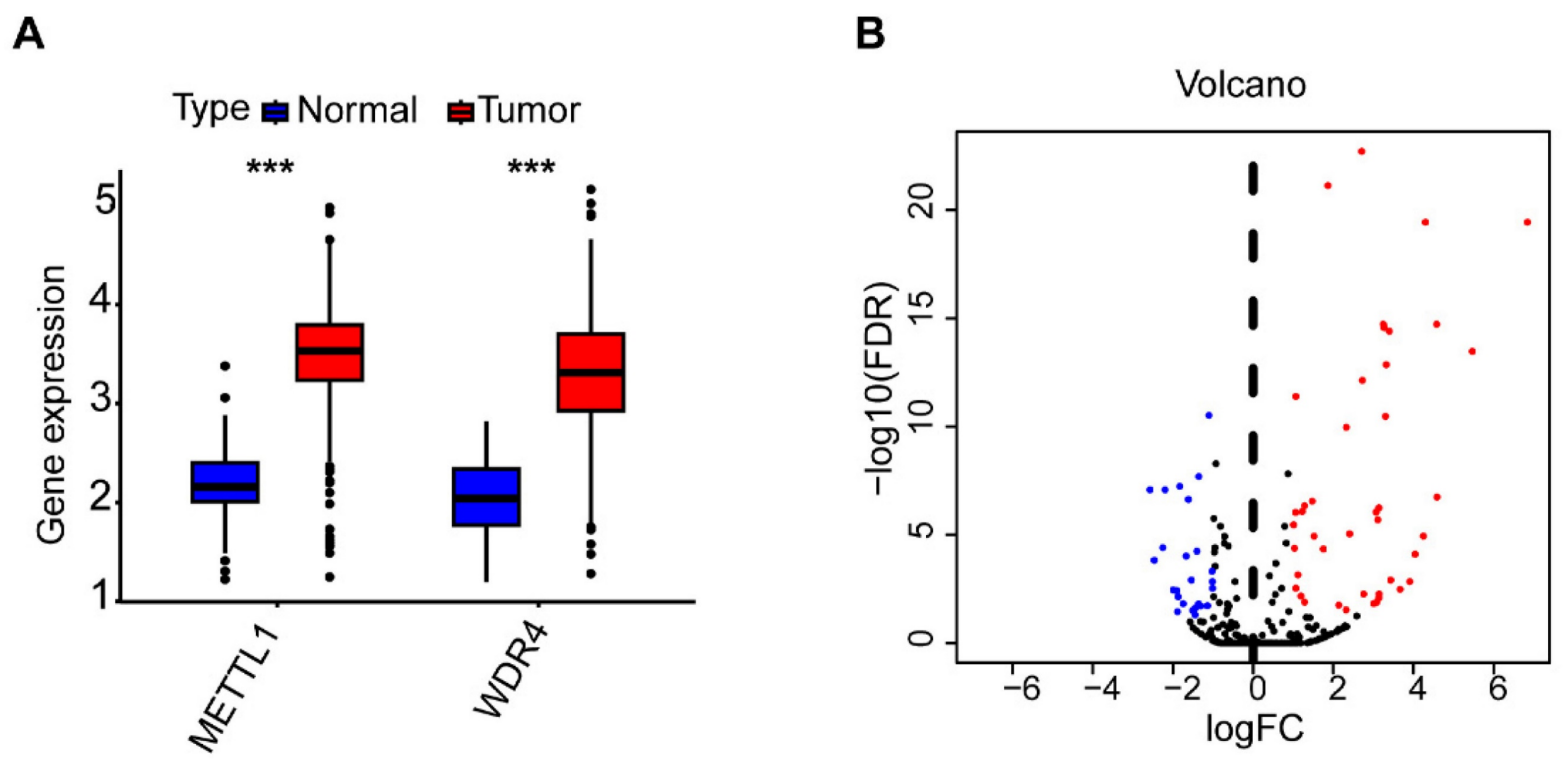
The expression levels of m7G methylation regulators and m7G-related miRNAs between tumor and normal samples in TCGA CC cohort. (**A**) the expression difference of METTL1 and WDR4 between tumor and normal samples; (**B**) The volcano of differentially expressed m7G-related miRNAs between tumor and normal samples.

A flowchart of our study is shown in Figure 1. The expressions of 2 m7G-related genes (METTL1 and WDR4) were significantly higher in CC tissues compared to normal tissues (Figure 2A), indicating their potential involvement in the pathogenesis of CC. Subsequently, we identified the potential miRNAs related to these two m7G-related genes employing the TargetScan software, which led to the selection of 792 m7G-related miRNAs for further analysis. To identify miRNAs potentially implicated in the development of CC, we compared the expression of 792 predicted miRNAs between 457 CC patients and 8 normal samples. A total of 42 DE-miRNAs with FDR < 0.05 and |log FC| ≥ 1 were obtained, of which 26 being up-regulated and 16 being down-regulated (Figure 2B). The top 30 most DE-miRNAs were used to generate a heatmap, which is presented in Supplementary Figure S1.

### Construction and verification of the prognostic signature based on m7G-related miRNAs

**Figure 3:**
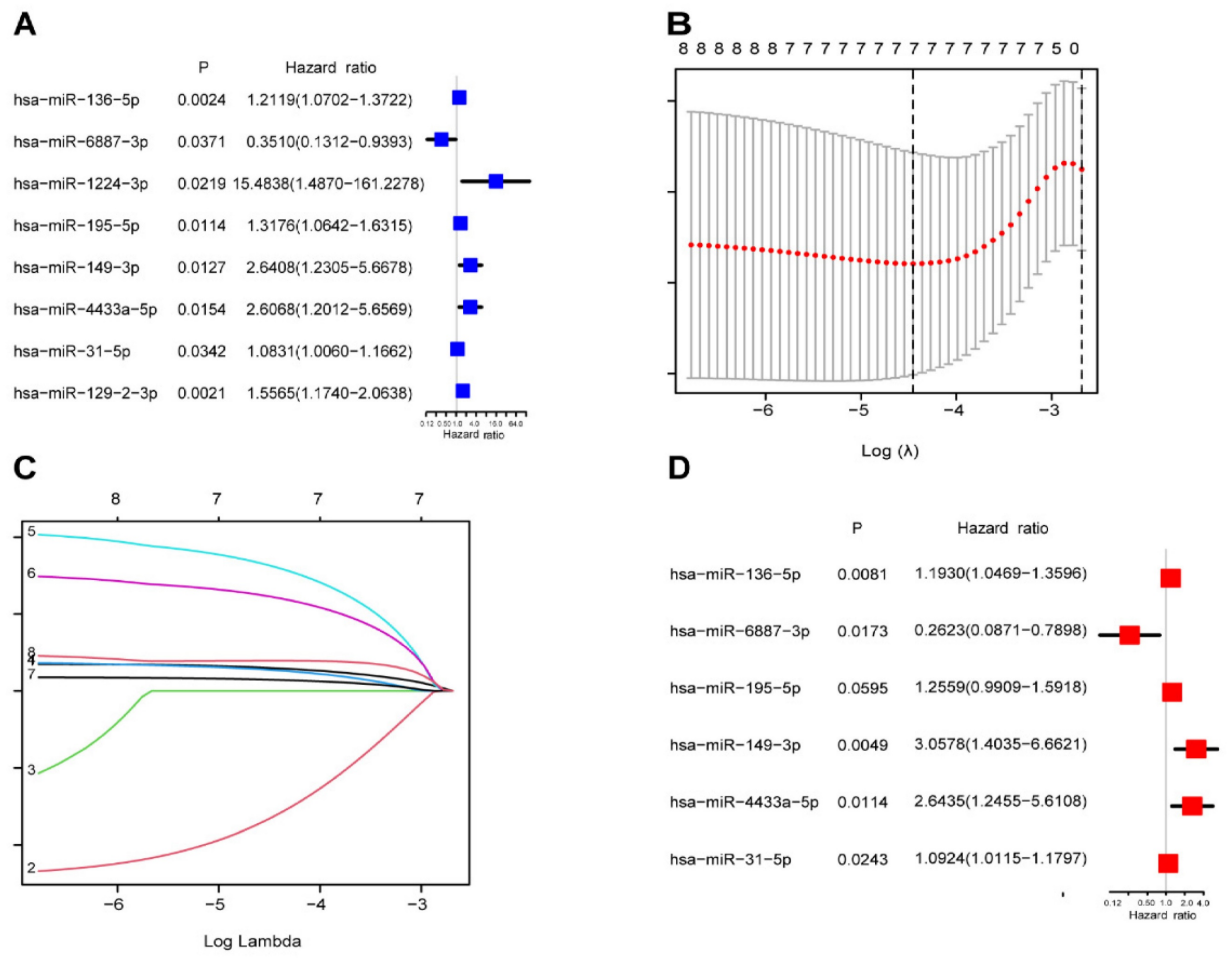
Identification of the prognostic m7G-related miRNAs. (**A**) The univariate Cox regression analysis of eight m7G-related DE-miRNAs; (**B**) The optimal λ selection by 10 cross validated partial likelihood deviance of the LASSO regression; (**C**) The LASSO coefficient profiles of seven prognostic m7G-related DE-miRNAs; (**D**) Forest plot summary of the HR values of seven m7G-related DE-miRNAs.

**Figure 4:**
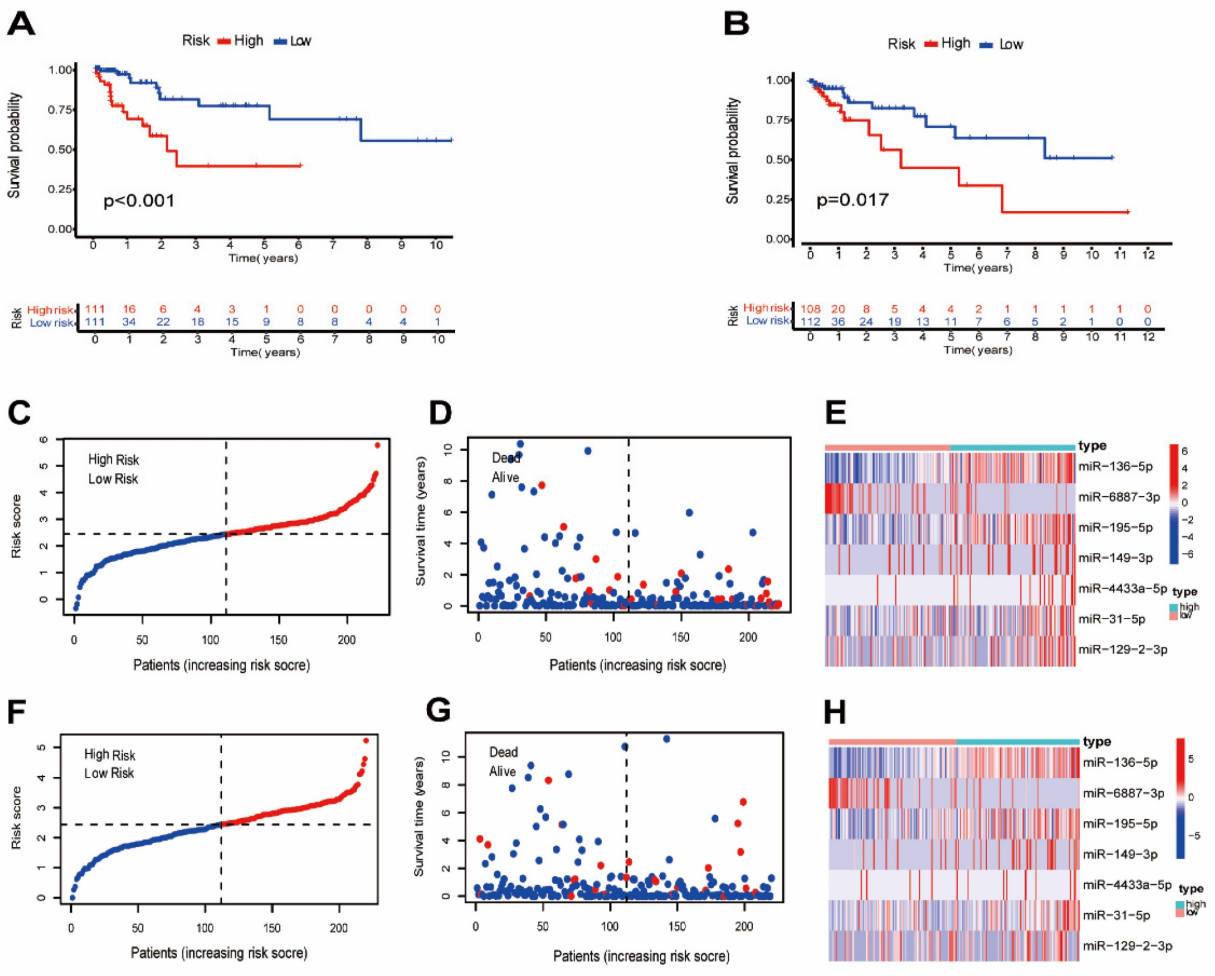
Evaluation of the prognostic m7G-related miRNAs. (**A,B**) Kaplan-Meier curves of different risk groups in the training and testing sets; (**C,F**) The distribution of risk scores ordered from low to high in the training and testing sets; (**D,G**) The distribution of survival time and survival state in the training and testing sets; (**E,H**) Heatmap of 7 risk miRNAs expression levels in the training and testing sets.

**Figure 5:**
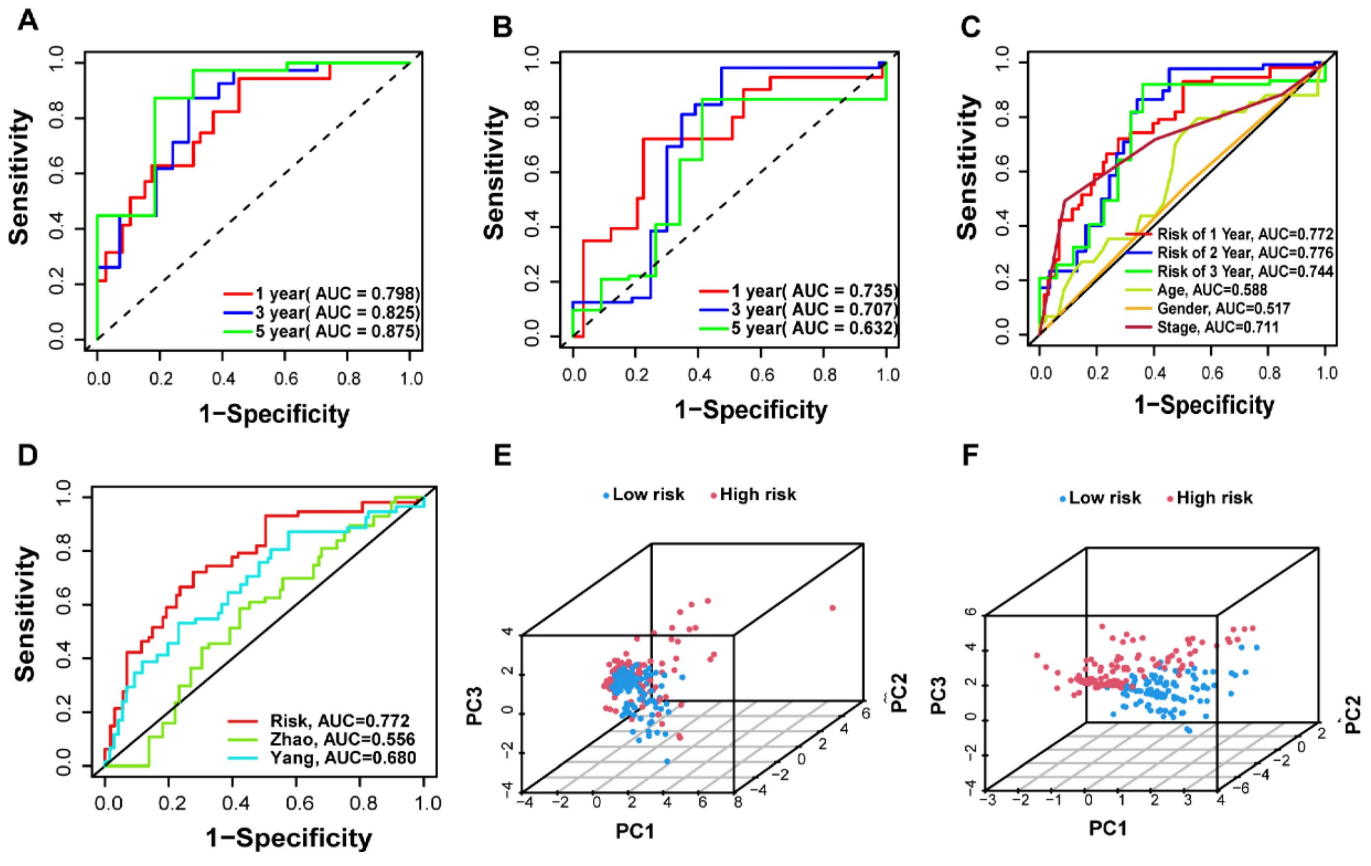
Validation of the prognostic m7G-related miRNAs signature. (**A-B**) The AUC curves to predict the sensitivity and specificity of 1-, 3- and 5-year survival according to the riskScore in training and testing sets; (**C**) Comparison of the AUCs of the riskScore at 1-, 3- and 5-year and clinical features; (**D**) ROC curves of different risk models; (**E-F**) Principal component analysis separated CC patients into high- and low-risk groups in the training and testing sets.

A total of 442 CC samples were randomly divided in a 1:1 ratio, with 222 samples allocated to the training set and 220 samples to the testing set. Table 1 presents the clinical characteristics of both patient groups, revealing no significant differences between the two groups. To investigate the prognostic significance of the 42 m7G-related DE-miRNAs, we performed univariate Cox regression analysis on the training set and determined eight candidate prognostic miRNAs for further evaluation (Figure 3A). Next, we applied LASSO analysis based on these eight miRNAs to remove overfitting genes from the model (Figure 3B,C). Following the minimum standard, a prognostic signature consisting of seven key m7G-related miRNAs was constructed (Figure 3D, Supplementary Table S1). The risk score for each patient was derived using the following calculation: riskScore = (0.1486 × miR-136-5p expression) + (-0.8152 × miR-6887-3p expression) + (0.1353 × miR-195-5p expression) + (0.8084 × miR-149-3p expression) + (0.6097 × miR-4433a-5p expression) + (0.0724 × miR-31-5p expression) + (0.1966 × miR-129-2-3p expression). The Kaplan-Meier (K-M) survival curves demonstrated a significant association between higher risk scores and worse prognosis, as demonstrated in Figure 4A,B. Additionally, there was a concurrent increase in both the death rate and the high-risk ratio as the risk scores increased (Figure 4C,D,F,G). The heatmap showed that miR-136-5p, miR-195-5p, and miR-31-5p were remarkably overexpressed in the high-risk group, indicating that these miRNAs could be poor prognostic predictors (Figure 4E,H). Moreover, the ROC analysis demonstrated the exceptional predictive capability of the prognostic signature based on m7G-related miRNAs for 1-, 3-, and 5-year outcomes. The AUC values were 0.798, 0.825, and 0.875 in the training set, and 0.735, 0.707, and 0.632 in the testing set, respectively (Figure 5A,B). Furthermore, we conducted a comparative analysis between the prognostic signature and other clinical features. The results revealed that the prognostic signature achieved superior performance in predicting 1-year outcomes, with the AUC value of 0.772. In contrast, conventional clinical features such as age (AUC = 0.588), gender (AUC = 0.517), and stage (AUC = 0.711) exhibited comparatively lower predictive accuracy (Figure 5C). We also compared the AUC values of our risk model in this manuscript with those of two other previously published miRNA prediction models for colon cancer. The results showed that the AUC value of our risk model (AUC=0.772) was significantly higher than that of the other two models (Zhao, AUC=0.556; Yang, AUC=0.680), suggesting that our approach can accurately predict the prognosis of patients with colon cancer and has high clinic efficacy (Figure 5D)^23^, ^24^. The results of PCA indicated that the high- and low-risk groups were well-separated along different directions (Figure 5E,F). Collectively, these findings provide evidence that the prognostic signature utilizing m7G-related miRNAs may serve as a reliable predictor for patients with CC.

### Relationship between the m7G-related miRNA signature and clinical features

**Figure 6:**
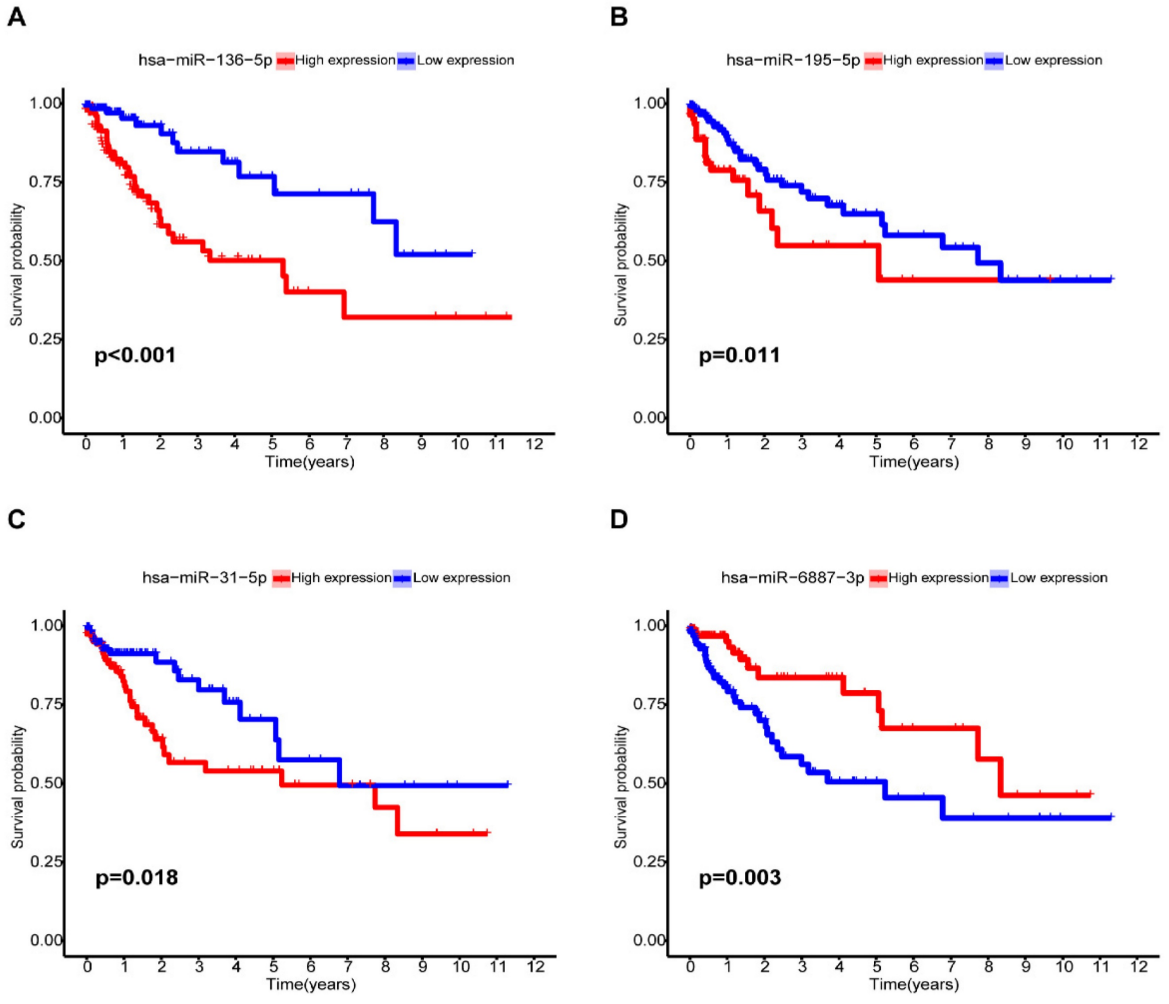
Kaplan-Meier analysis of miRNAs for the overall survival in CC patients. (**A**)miR-136-5p; (**B**) miR-195-5p; (**C**) miR-31-5p; (**D**) miR-6887-3p.

**Figure 7:**
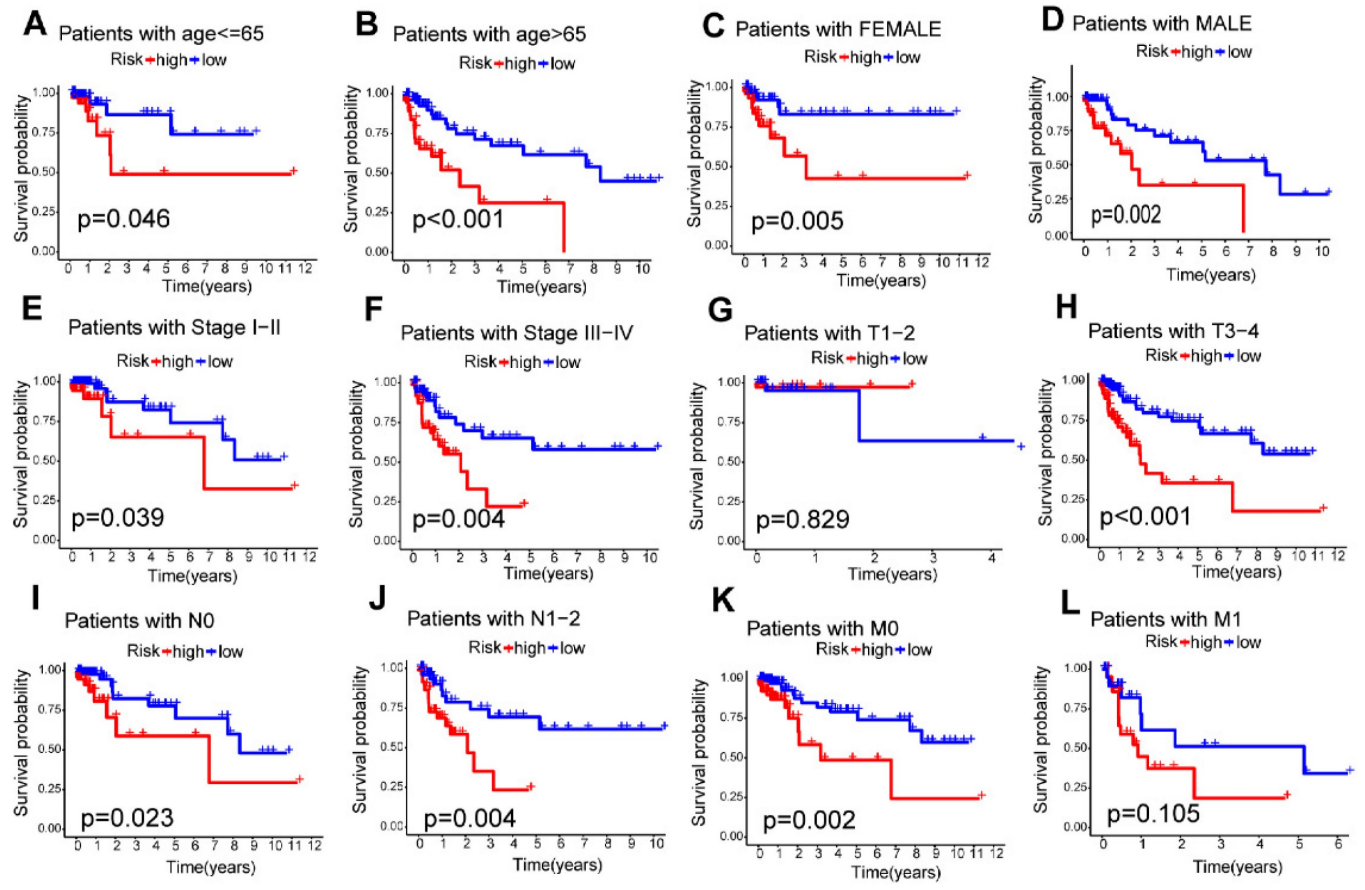
Relationship between the m7G-related miRNA signature and clinical features. (**A-L**) Kaplan-Meier curves for patients with different clinical features; (**M-R**) Distribution of risk scores stratified by different clinical subgroups.

Among the seven risk miRNAs analyzed, three miRNAs, namely miR-136-5p, miR-195-5p, and miR-31-5p, were identified as independent prognostic risk factors, while miR-6887-3p was discovered to be an independent prognostic protective factor (Figure 6A-D). We conducted stratified survival analysis to assess the predictive capability of the signature for OS across various clinical subgroups, including age, gender, pathological stage, stage T, stage N, and stage M. The K-M survival analyses revealed that the low-risk group exhibited significantly improved OS compared to the high-risk group across different age groups (≤ 65, > 65), gender (male, female), pathological stages (stage I-II, stage III-IV), and stage N (N0, N1-2) (p < 0.05). The signature was found to be suitable for patients with M0 but not for M1 (p=0.105). In addition, the prognostic signature had extreme significance for patients with T3-4 (p < 0.001), but not for patients with T1-2 (p = 0.829) (Figure 7A-L). Furthermore, our analysis revealed a significant association between the risk score and various clinical features, including age and TNM stages, as well as pathological stage (Supplementary Figure S2). These findings solidify the significance of the risk score derived from our prognostic signature as a robust predictor for the prognosis of CC patients.

### Development of a prognostic nomogram

**Figure 8:**
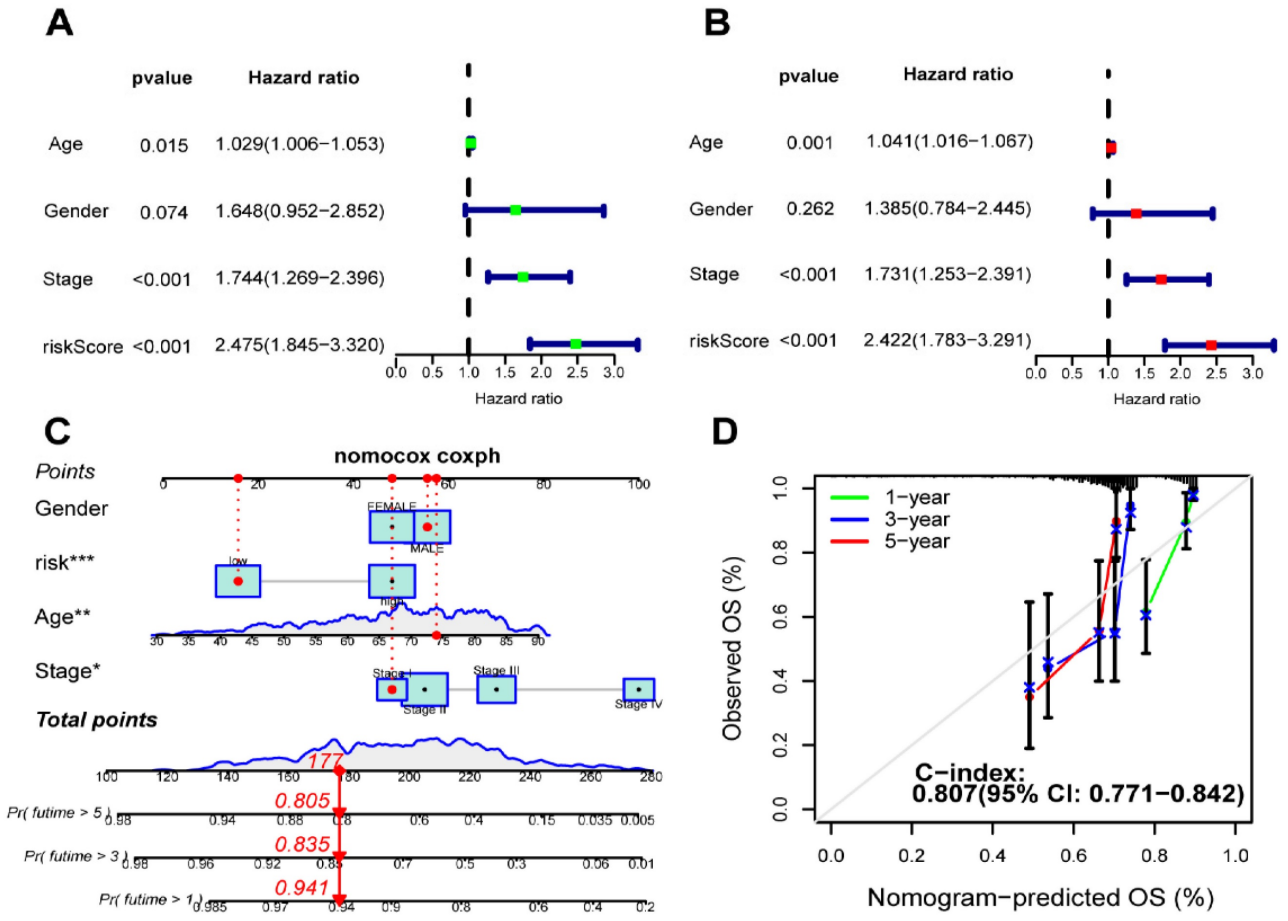
Establishment and validation of a nomogram. (**A**) The correlation of clinical features and riskScore was analyzed by univariate Cox regression analysis related to overall survival; (**B**) The correlation of clinical features and riskScore was analyzed by multivariate Cox regression analysis with overall survival; (**C**) A nomogram based on clinical features and riskScore to predict 1-, 3- and 5-year survival; (**D**) Calibration curve of the nomogram.

We performed univariate and multivariate Cox regression analyses to determine whether our signature is independent of other clinical prognostic factors affecting patient outcomes. Following the univariate regression analysis, factors associated with OS in CC patients were identified. Subsequently, multivariate Cox analysis exhibited that riskScore (p < 0.001, HR = 2.422), age (p = 0.001, HR = 1.041) and stage (p < 0.001, HR = 1.731) emerged as independent variables significantly correlate with OS (Figure 8A,B). Furthermore, we constructed a nomogram that integrates both the prognostic signature and clinicopathological features to assess the OS of CC patients (Figure 8C). The C-index was 0.807 (95%CI = 0.771-0.842). The calibration curves demonstrated a strong concordance between the predicted and observed 1-year, 3-year, and 5-year OS rates, confirming the high reliability of the prognostic nomogram (Figure 8D).

### Impact of the risk model of m7G-related miRNAs on immune infiltration

**Figure 9:**
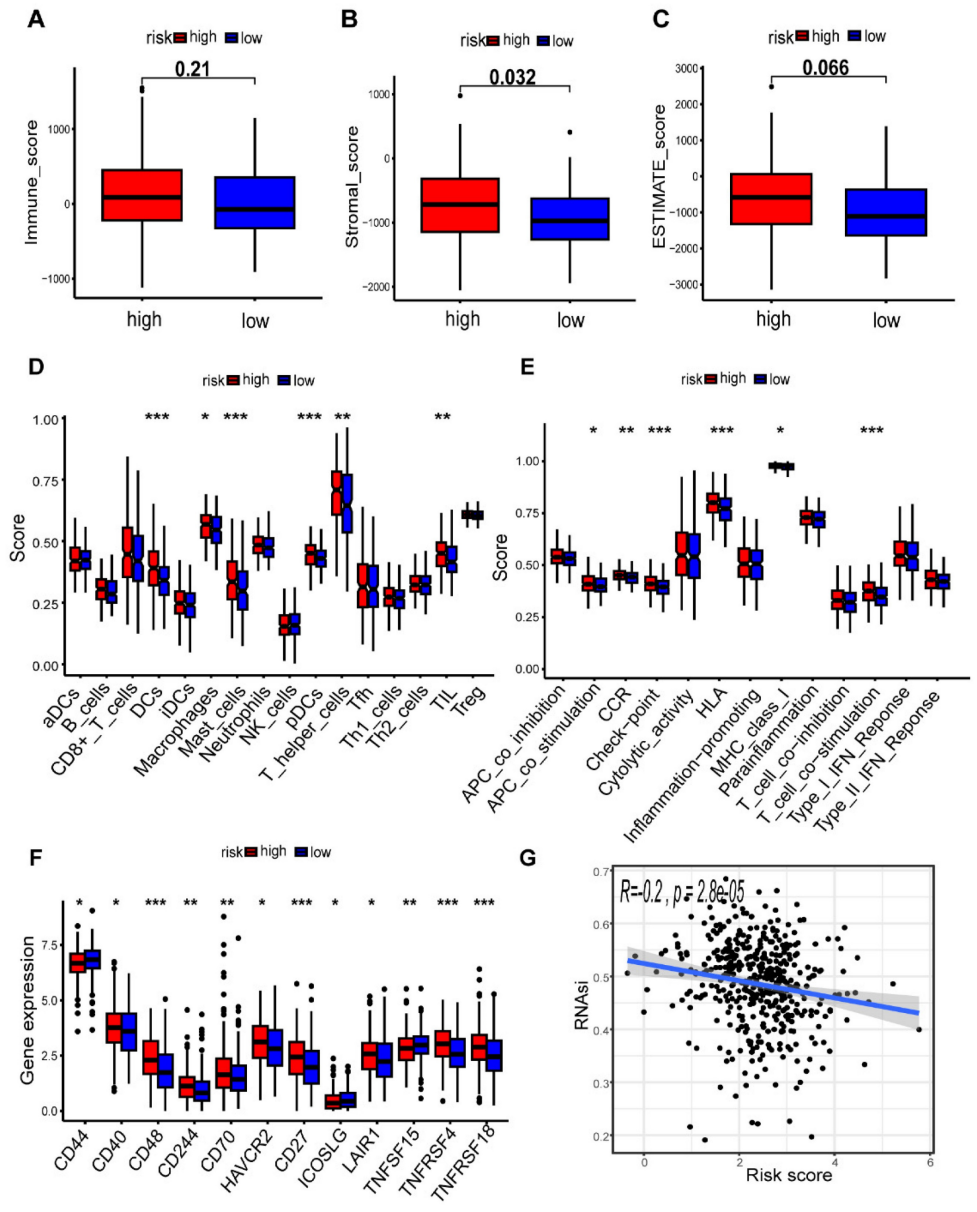
Comparison of the tumor microenvironment (TME) and immune features in the two risk groups. (**A-C**) Analysis of differences in TME scores between different risk groups; (**D**) Correlation analysis of immune cells; (**E**) Correlation analysis of immune functions; Comparison of 16 immune cell scores (**F**) and 13 immune-related function scores (**G**) between high- and low-risk groups; (**H**) Expression levels of common immune checkpoints in different groups; (**I**) Cancer stemness feature analysis.

In order to assess the impact of the risk-level model on TME, we utilized the “ESTIMATE” algorithm to obtain TME-related scores, which included stroma score, immune score, and estimate score. Our findings revealed a significant association between the high-risk group and elevated stromal scores, suggesting a potential correlation between risk model and TME in patients with CC (p < 0.05) (Figure 9A-C). We further investigated the association between immunocytes and immunofunctions, as depicted in Supplementary Figure S3. Next, we performed an analysis of the diversities in 29 types of immune-associated gene sets using the ssGSEA algorithm. Interestingly, we observed significant differences in the scoring of immunocytes, such as Macrophages, Mast cells, tumor-infiltrating lymphocytes (TIL) and other immune cells, between the high- and low-risk groups (Figure 9D). Additionally, there were remarkable disparities in the scores of immunofunctions, such as HLA, T cell co-stimulation, Check-point, CCR and other immune-related pathways (Figure 9E). In summary, our results suggest that the levels of immune infiltration vary considerably between the two risk groups, with the high-risk group being interrelated with increased immune/inflammation activity. In addition, we discovered that the majority of immune checkpoints had higher expression in the high-risk group, which suggests that patients in this group may have a better treatment prospect for targeted therapy with a more favorable prognosis (Figure 9F). Additionally, our correlation analysis revealed that the mRNAsi score was negatively association with riskScore (r = -0.2, p = 2.8e-05), indicating that patients in the high-risk group might exhibit lower cancer stemness (Figure 9G). We further assess the effect of METTL1 and WDR4 on the immune microenvironment in colon cancer by ssGSEA algorithm. The results revealed that METTL1 and WDR4 showed a negative correlation with the immune infiltrating cells except CD8+ T cells, DCs, mast cells, and pDCs (Supplementary Figure S4). The expression of METTL1/WDR4 was both significantly negatively correlated with the levels of immune cell infiltration of B cells, Macrophages, Neutrophils, NK cells, TIL, and regulatory T (Treg) cells (|r| ≥ 0.2, p < 0.001).

**Figure 10:**
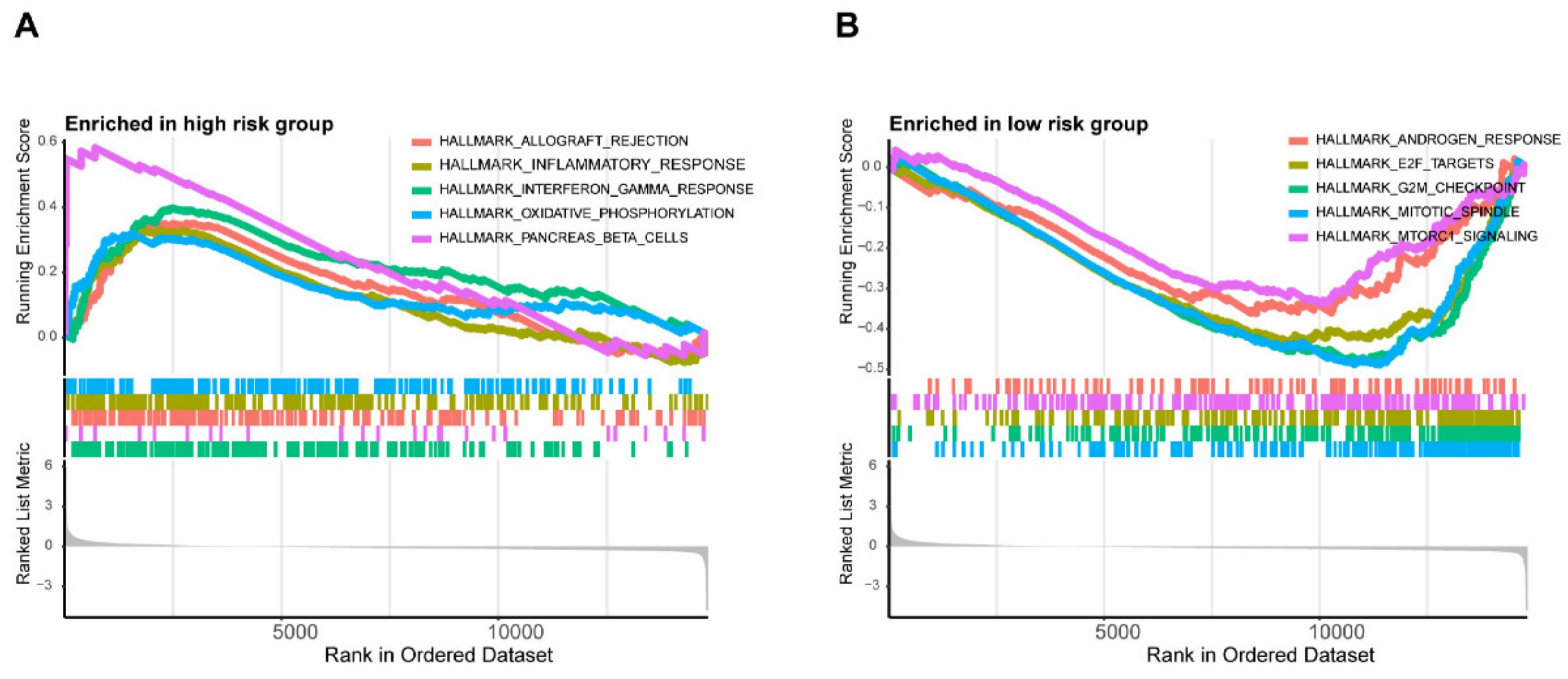
Differences in the biological pathways involved in the two risk groups. GSEA determines the first 5 hallmarks enriched in the high-risk group (**A**) and the low-risk group (**B**).

In addition, the enrichment scores of most immunocytes including Macrophages, Neutrophils, NK cells in the METTL1 high expression group were markedly lower than those in the METTL1 low expression group (all p < 0.001), which was consistent with WDR4 (Supplementary Figure S4). These results indicated that METTL1/WDR4 overexpression may affect the progression and prognosis of colon cancer by regulating the levels of infiltrating immune cells. The results of GSEA showed the first five hallmark pathways enriched in the high-risk group included allograft rejectio, inflammatory response, interferon-gamma response, oxidative phosphorylation, and pancreas-beta cells. Conversely, the low-risk group was involved with androgen response, E2F targets, the G2/M checkpoints, the mitotic spindle and mTORC1 signaling (Figure 10A,B).

### Identification of prognostic genes associated to m7G-related signature and immunity

**Figure 11:**
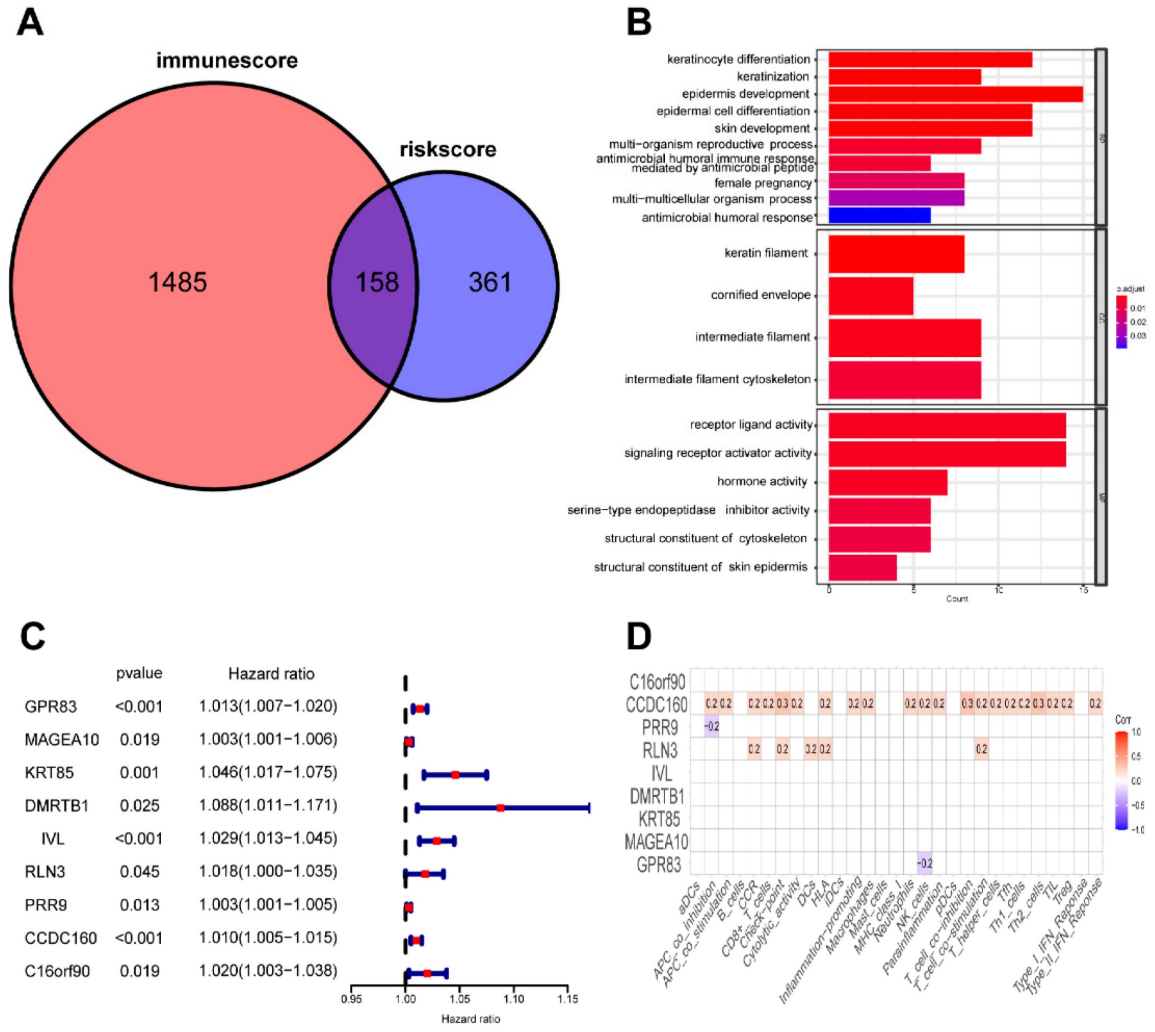
Identification of mRNAs related to riskScore and immunity. (**A**) 158 mRNAs associated with riskScore and immune infiltration; (**B**) GO enrichment analysis of 158 risk-immune-related mRNAs; (**C**) The univariate Cox regression analysis of 158 risk-immune-related mRNAs; (**D**) Correlation of prognostic risk-immune-related mRNAs and immune infiltration.

To evaluate the risk model's value at various molecular levels, we conducted differential analysis of the CC dataset, which resulted in 519 DE-mRNAs between two risk groups. In addition, we determined 1643 differentially expressed mRNAs based on the ESTIMATE immune score in the high-immunoscore and low-immunoscore groups. The intersection of these two sets of DE-mRNAs yielded 158 genes related to both risk levels and immune status (Figure 11A). Moreover, we annotated the function of these 158 genes based on Gene Ontology (GO) terms and found that most of them were implicated in tumorigenesis and immune-related signaling pathways (Figure 11B). We further identified nine prognostic risk-immune-related genes that were all predictive of poor prognosis through univariate Cox analysis (Figure 11C). Ultimately, Pearson correlation analysis revealed a positive association between CCDC160 and RLN3 genes and immune filtration (Figure 11D).

### Expression validation of prognostic miRNAs in CC Cells

**Figure 12:**
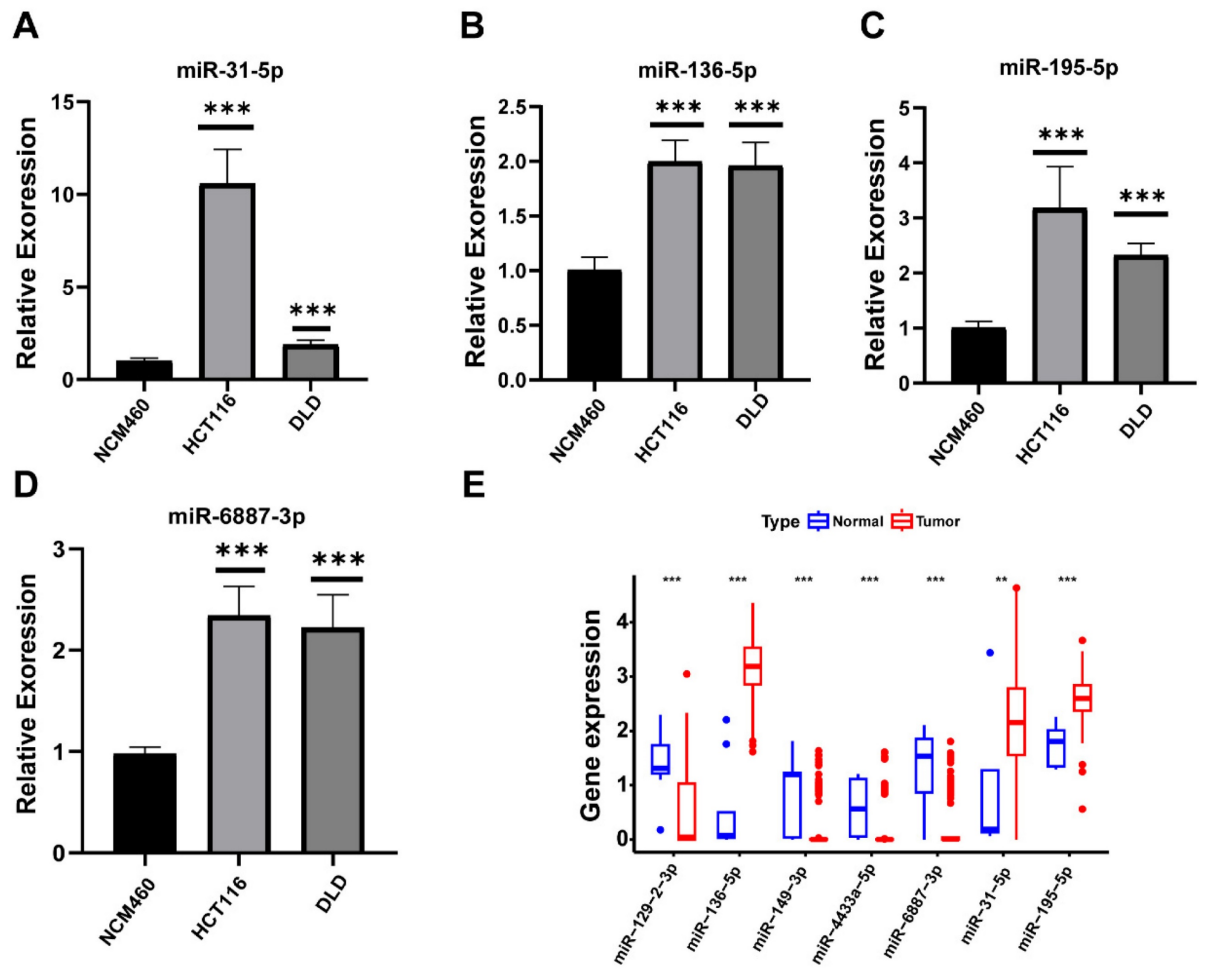
Relative expression of miRNAs. (**A**)miR-136-5p; (**B**) miR-195-5p; (**C**) miR-31-5p; (**D**) miR-6887-3p; (**E**) Differential expression of miRNAs in TCGA.

Compared to normal cells, the expression levels of four prognostic miRNAs including miR-31-5p, miR-136-5p, miR-195-5p, and miR-6887-3p were relatively higher in tumor cell lines respectively (Figure 12A-D). The expression trends of miR-136-5p, miR-195-5p, and miR-31-5p were consistent with the predict results (Figure 12E).

## Discussion

Colon cancer (CC) is a prevalent malignancy affecting the digestive system, frequently resulting in unfavorable outcomes for patients. Despite efforts to develop prognostic biomarker-based models, current approaches have proven insufficient in meeting the demands of clinical treatment and prognosis evaluation. Therefore, it is imperative to explore novel prognostic indicators or develop effective therapeutic targets. Recent studies have demonstrated that m7G modification play a significant role in the development and advancement of diverse cancer types, such as acute myeloid leukemia, breast cancer, and kidney renal clear cell carcinoma^25^, ^26^, ^27^. Moreover, mounting evidence suggests that METTL1/WDR4 complex is intricately linked with various tumors via the m7G modification. Given the significant role of dysregulated miRNAs in the pathogenesis of CC and their relation to response to chemoradiotherapy^28^, ^29^, circulating miRNAs have emerged as a promising non-invasive biomarker for diagnosis and prognosis^18^. Therefore, the establishment of an m7G-related miRNA signature holds great clinical value and may provide critical insights into developing novel therapeutic strategies for CC.

In our study, we have identified 42 differentially expressed miRNAs associated with m7G modification among a total of 792 m7G-related miRNAs between CC and normal samples. Univariable Cox analysis indicated that eight differentially expressed miRNAs associated with m7G modification had prognostic significance. Further, we successfully set up a novel prognostic signature comprising seven differentially expressed miRNAs associated with m7G modification using LASSO Cox analysis. The K-M curves clearly demonstrated that patients belonging to the high-risk group exhibited significantly shorter survival times compared to those in the low-risk group. The AUC of the signature indicated good accuracy in predicting 1-, 3-, 5-year survival rates of CC patients. Moreover, our signature showed promising independence in forecasting the prognoses of CC patients. These findings were further validated in the test cohort. The potency of our nomogram was also confirmed by calibration plots. In addition, we utilized the ESTIMATE database of CC patients to identify 158 differentially expressed risk-immune-related mRNAs based on the risk score and the immunoscore. These genes were subsequently analyzed to understand their biological functions and pathways associated with risk score and immunity. Our findings show that the majority of these genes are related to oncogenesis pathways. In addition, using univariate Cox regression analysis, we identified nine prognostic risk-immune-related DE-mRNAs that may influence CC progression at both the m7G and immune levels. CCDC160 and RLN3 was the gene most relevant to immune cells and function in CC. Overall, our findings strongly suggest that the developed signature holds great potential as a valuable tool to make informed decisions and provide appropriate guidance.

The remarkable success of immunotherapy has brought about a paradigm shift in the management of cancer patients. Novel immune-based therapeutic approaches, such as adoptive cell therapy utilizing tumor-infiltrating lymphocytes and immune checkpoints inhibitors (ICIs), are currently at the forefront of cancer research and hold great promise^30^. Upon conducting a differential TME analysis, we observed that the high-risk group consistently exhibited higher scores compared to the low-risk group, indicating a lower tumor purity and a greater presence of immune and stromal cells within the TME. In addition, our findings revealed significantly elevated infiltration levels of immune-related gene sets within the high-risk group, indicating heightened immune activity amongst patients in this cohort. We observed that Macrophages and mast cells were greatly enriched in the high-risk group. Tumor-associated macrophages (TAMs) are widely known to create a favorable milieu for tumor progression within the TME. They contribute to various processes, including facilitating tumor cell growth, promoting epithelial-mesenchymal transition (EMT), and suppressing immune responses. Studies have demonstrated a close association between M2-like TAMs and poor prognosis in CRC, which is regulated by miR-195-5p/NOTCH2 axis^31^. Additionally, Xu *et al.*^32^ proposed that mucosal mast cell activation recruits and modulates the CD11b+Gr1+ cells to promote CRC growth. Based on our findings, it is possible that the poorer survival outcomes observed in high-risk patients could be attributed to heightened levels of pro-tumor immunity. Our study additionally revealed significantly up-regulation of almost all immune checkpoints in high-risk patients, possibly indicating that this group would benefit from immunotherapy with targeted ICIs. We also assessed the effect of METTL1 and WDR4 on the immune microenvironment in colon cancer. The expression of METTL1/WDR4 was both significantly negatively correlated with the levels of most immune cell infiltration. Consistently, it was reported that METTL1 expression increased after radiofrequency ablation of recurrent HCC, which was accompanied by decreased CD8+ T-cell infiltration and increased infiltration of polymorphonuclear myeloid-derived suppressor cells (PMN-MDSCs) 33. Liu et.al recently revealed that METTL1-mediated m7G modification significantly regulates PMN-MDSCs accumulation in the immune microenvironment and intrahepatic cholangiocarcinoma progression through targeting CXCL8 in humans and Cxcl5 in mice 34. Furthermore, WDR4 negatively regulates PML expression to enhance lung cancer development by creating a pro-metastatic and immunosuppressive status, which may be helpful for potential future treatments in lung cancer patients 35.

Relaxin‑3 (RLN3), a gene associated with immune cells and functions, was discovered to have a positive correlation in our study. RLN3 is the ancestral peptide of the human relaxin (RLN) subclass of the insulin superfamily. It was reported to inhibit fibroblast activation after binding to its primary receptor RXFP1 (relaxin family peptide receptor type 1)^36^. Numerous studies have demonstrated that RLN can modulate the tumor microenvironment by stimulating various immune cells, thereby influencing tumor development and progression. Zhou *et al.* reported that RLN delivery induces an increase in intratumoral F4/80+CD206+ macrophages derived from Ly6C+ monocytes, promoting fibrosis resolution and cytotoxic T cell infiltration. Furthermore, RLN gene delivery synergistically inhibits tumor growth by enhancing T cell-mediated tumor cell killing and macrophage phagocytosis in conjunction with PD-L1 blockade^37^. Hu *et al.* demonstrated that a combination therapy involving RLN, FOLFOX, and IL-12 successfully stimulates central memory T cells, achieving long-term survival in invasive colorectal cancer liver metastasis models; notably, this approach resulted in complete tumor remission in 50% of the mouse models, providing durable protection against tumor recurrence^38^. Currently, there is limited research on CCDC160; however, our study found that CCDC160 is positively correlated with immune cells and their functions in colon cancer, suggesting that CCDC160 may represent a novel target in this context. Future research could further investigate the role of CCDC160 in the pathogenesis of colon cancer.

Apart from RLN3 and CCDC160, the role of m7G-related miRNAs in immune microenvironment cannot be disregarded. Abnormal glucose metabolism is one of the key hallmarks of cancer. Cancer cells adapt to the changes in the tumor microenvironment by shifting their metabolism from oxidative phosphorylation to glycolysis. Studies have shown that in colorectal cancer, miR-149-3p targets pyruvate dehydrogenase kinases 2 (PDK2) to promote 5-fluorouracil (5-FU)-induced apoptosis and reduce glucose metabolism within the tumor microenvironment^39^. Furthermore, research has also indicated that miR-149-3p inhibits regulatory T cell differentiation and immune escape in esophageal cancer by targeting FOXP3^40^. Anti-PD-(L)1 antibodies, as representative ICIs, have ushered in new hope for the treatment of colorectal cancer. The microsatellite instability (MSI) status can significantly influence the tumor microenvironment in colorectal cancer patients from multiple perspectives. Compared to microsatellite stable (MSS) and MSI-low (MSI-L) colorectal cancers, microsatellite high (MSI-H) colorectal cancers exhibit greater immune cell infiltration, higher expression of immune-related genes, and increased immunogenicity. Consequently, patients with MSI-H tumors derive significant benefits from ICI therapy. Research has shown that miR-31 mediates the downregulation of SATB2 expression by binding to its 3'-UTR. A cohort study observed a strong negative correlation between microsatellite instability in colorectal cancer and SATB2 expression levels^41^. Additionally, the upregulation of miR-195-mediated PD-1/PD-L1 immune checkpoint blockade effectively stimulates T cell infiltration, activates natural killer (NK) cells, promotes dendritic cell maturation, and enhances cytotoxic T lymphocyte (CTL) killing activity^42^. These immune analyses have established a theoretical framework for future research and have yielded valuable insights into tumor progression, immune status and individualized immunotherapy for patients with CC.

Subsequently, we conducted GSEA to investigate the underlying biological processes associated with the two distinct subgroups. Our results unveiled significant enrichment of multiple inflammation/immunity-related pathways specifically within the high-risk group, which might potentially relate to the increased immune activity observed in this subgroup. Conversely, the low-risk group exhibited an enrichment of malignant functional features, such as mTORC1 signaling and E2F targets. Notably, mTORC signaling is recognized for its pivotal role in tumorigenesis, particularly in apoptosis regulation, cell cycle control, and cancer cell proliferation. Gulhati *et al.*^43^ reported that elevated mTORC1 and mTORC2 activity is involved in the regulation of EMT and the metastasis of CRCs through the signaling pathways involving RhoA and Rac1. Numerous studies have underscored the significance of the E2F family in the development of colon cancer. For instance, E2F1 has been shown to enhance the expression of c-Myc and p14ARF, leading to apoptosis in colon cancer cells. Additionally, E2F2 has emerged a prospective molecular biomarker for assessing colon carcinogenesis^44^. Based on the findings, we postulated that targeting the mTORC signaling pathway molecules and the E2F family through m7G methylation modification may regulate the differential TME between the two groups in CC.

We performed additional analysis to investigate the influence of seven miRNAs expression on the prognosis of patients suffering from CC. Our in-silico analysis and qRT-PCR results demonstrated that miR-136-5p and miR-31-5p were both highly expressed in tumor tissue. This higher expression level was associated with a lower survival rate than the higher expression group, which are consistent with the discovery of numerous researchers^45^, ^46^. Furthermore, previous studies have revealed that miR-136-5p enhances the expression of SH2B1 by interacting with hsa_circ_0136666, thus promoting the proliferation and invasion of CRC^47^. Mi *et al.*^48^ reported that the upregulation of miR-31-5p contributed to CC progression through its targeting of TNS1 and modulation of immune infiltration. Our study has been bolstered by these functionally verified results, which provide additional support for our findings. Similarly, our integral analysis results showed that miR-195-5p was expressed at significantly higher levels in CC tissues and identified as a prognostic risk factor. However, our research differs from earlier studies regarding the function of miR-195-5p. Wang *et al.*^49^ reported that miR-195-5p could act as a tumor suppressor in CRC and impair tumorigenesis and the stemness of colon cancer cells through its direct targeting of minichromosome maintenance marker 2 (MCM2). Thus, further exploration of the specific molecular function of miR-195-5p in CC is necessary. Furthermore, our experimental results showed that miR-6887-3p was highly expressed in tumor cell lines, which was inconsistent with the results of differential expression analysis and the survival analysis. This suggested that the role of miR-6887-3p in colon cancer may be complex. We also found that the other model miRNAs are tightly bound up with tumorigenesis and development. For example, targeting DDX17 via miR-149-3p has been shown to suppress metastasis and EMT of CRC cells^50^. Although miR-4433a-5p and miR-129-2-3p have not yet been reported in CC studies, they have been studied in other malignancies. For example, miR-129-2-3p has been demonstrated to facilitate the suppression of tumor growth in gastric cancer by targeting CDK6, a cell cycle-associated protein that plays a crucial role in the G1-S transition^51^. Studies have shown that miR-4433a-5p expression notably increased in papillary thyroid cancer (PTC) and could serve as a biomarker for PTC diagnosis^52^. Nonetheless, further investigations are required to uncover their specific functions. Considering the substantial contributions of the seven investigated miRNAs to the progression of CC and other cancers, the signature can be deemed dependable in predicting patients' prognoses. Our findings will also shed light on future CC research. Further studies are warranted to comprehensively investigate the underlying mechanisms and signaling pathways through which these miRNAs exert their effects in CC.

However, our study still had a few limitations that should be addressed. Firstly, our conclusions were solely drawn from the results of bioinformatic analysis conducted on publicly available databases. As such, the lack of an external database to validate our findings may have introduced inherent bias into our study. Therefore, future research efforts should focus on exploring additional sources of data to further confirm our results. To this end, further research should be employed to fully elucidate the functions of these m7G-related miRNAs and their interactions with m7G modification.

## Conclusion

Based on our knowledge, this study represents the first systematic investigation into the prognostic and immunological significance of m7G-related miRNA characteristics in colon cancer. We developed a novel prognostic signature based on m7G-related miRNAs for colon cancer patients, with the resulting risk scores demonstrating considerable potential as prognostic biomarkers. This model facilitates the identification of high-risk patients with poor survival rates, thereby enabling earlier and more proactive interventions. We have also introduced novel miRNA prognostic markers that have not been previously investigated in the context of colorectal cancer. In the future, these markers may serve as a basis for the development of new immunotherapeutic targets. Collectively, these findings provide critical evidence to support subsequent research endeavors aimed at elucidating the essential roles of m7G-related miRNAs in colon cancer and present promising pathways for the implementation of effective immunotherapy strategies for affected patients.

## Supplementary Materials

Supplementary figures and table.

## Figures and Tables

**Table 1:** Baseline clinical characteristics of CC patients in the TCGA cohort.

Covariates	Total set (n = 442)	Training set (n = 222)	Testing set (n = 220)	P-value
Age				
<=65	185 (41.86%)	89 (40.09%)	96 (43.64%)	0.5097
>65	257 (58.14%)	133 (59.91%)	124 (56.36%)	-
Gender				
Female	211 (47.74%)	106 (47.75%)	105 (47.73%)	1
Male	231 (52.26%)	116 (52.25%)	115 (52.27%)	-
Stage				
Stage-I	74 (16.74%)	36 (16.22%)	38 (17.27%)	0.0806
Stage-II	172 (38.91%)	80 (36.04%)	92 (41.82%)	-
Stage-III	120 (27.15%)	57 (25.68%)	63 (28.64%)	-
Stage-IV	65 (14.71%)	42 (18.92%)	23 (10.45%)	-
Unknown	11 (2.49%)	7 (3.15%)	4 (1.82%)	-
T				
T1-2	84 (19%)	40 (18.02%)	44 (20%)	0.5244
T3-4	350 (79.19%)	178 (80.18%)	172 (78.18%)	-
Tis	1 (0.23%)	0 (0%)	1 (0.45%)	-
Unknown	7 (1.58%)	4 (1.8%)	3 (1.36%)	-
N				
N0	258 (58.37%)	118 (53.15%)	140 (63.64%)	0.1143
N1	66 (14.93%)	34 (15.32%)	32 (14.55%)	-
N1a	15 (3.39%)	7 (3.15%)	8 (3.64%)	-
N1b	14 (3.17%)	5 (2.25%)	9 (4.09%)	-
N1c	2 (0.45%)	2 (0.9%)	0 (0%)	-
N2	61 (13.8%)	38 (17.12%)	23 (10.45%)	-
N2a	8 (1.81%)	5 (2.25%)	3 (1.36%)	-
N2b	11 (2.49%)	8 (3.6%)	3 (1.36%)	-
Unknown	7 (1.58%)	5 (2.25%)	2 (0.91%)	-
M				
M0	320 (72.4%)	154 (69.37%)	166 (75.45%)	0.3028
M1	65 (14.71%)	38 (17.12%)	27 (12.27%)	-
MX	50 (11.31%)	26 (11.71%)	24 (10.91%)	-
Unknown	7 (1.58%)	4 (1.8%)	3 (1.36%)	-

## Abbreviations

CC: Colon cancer
m7G: N7-methylguanosine
TME: tumor microenvironment
qRT-PCR: quantitative reverse transcriptase PCR
AUC: area under the curve
CRC: Colorectal cancer
EoCRC: Early-onset colorectal cancer
m6A: N6-methyladenosine
m5C: N5-methylcytosine
miRNA: microRNA
TCGA: The Cancer Genome Atlas
DE-miRNAs: differentially expressed miRNAs
LASSO: least absolute shrinkage and selection operator
PCA: principal component analysis
OS: overall survival
ROC: receiver operating characteristic
GSEA: single sample gene set enrichment analysis
DE-mRNAs: differentially expressed mRNAs
K-M: Kaplan-Meier
TIL: tumor-infiltrating lymphocytes
GO: Gene Ontology
ICIs: immune checkpoints inhibitors
TAMs: Tumor-associated macrophages
EMT: epithelial-mesenchymal transition
PMN-MDSCs: polymorphonuclear myeloid-derived suppressor cells
RLN3: Relaxin‑3
RXFP1: relaxin family peptide receptor type 1
PDK2: pyruvate dehydrogenase kinases 2
MSI: microsatellite instability
MSS: microsatellite stable
NK: natural killer
CTL: cytotoxic T lymphocyte
MCM2: minichromosome maintenance marker 2
PTC: papillary thyroid cancer
