# Supplementary material for: A novel m7G-related miRNA prognostic signature for predicting clinical outcome and immune microenvironment in colon cancer
Source: J Cancer. 2024 Oct 7;15(18):6086–102. doi: 10.7150/jca.99173 (PMC11493006; doi:10.7150/jca.99173)
Supplement: Supplementary file 1 — Supplementary figures and table. [file jcav15p6086s1.pdf]

Supplementary Materials

Figures:

Figure S1. Heatmap of the top 30 most differentially expressed miRNAs expression levels in each sample;

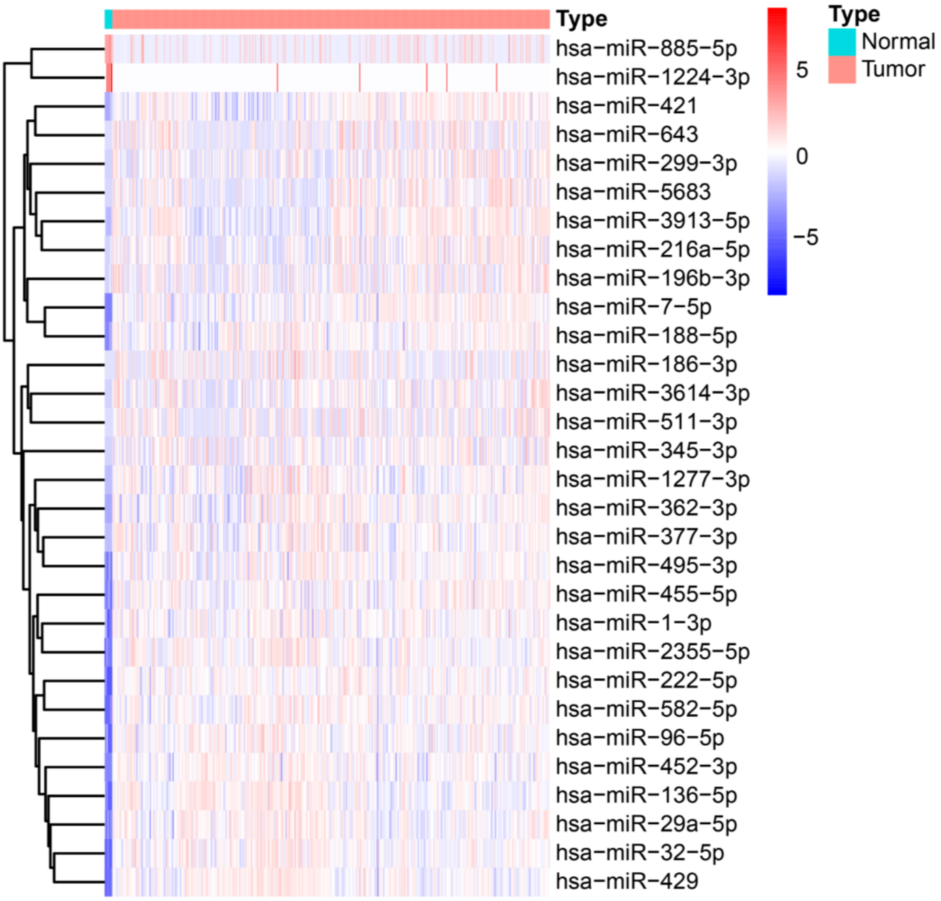

Figure S2. Distribution of risk scores stratified by different clinical subgroups. Age (A), gender (B), stage (C), T stage (D), N stage (E) and M stage (F);

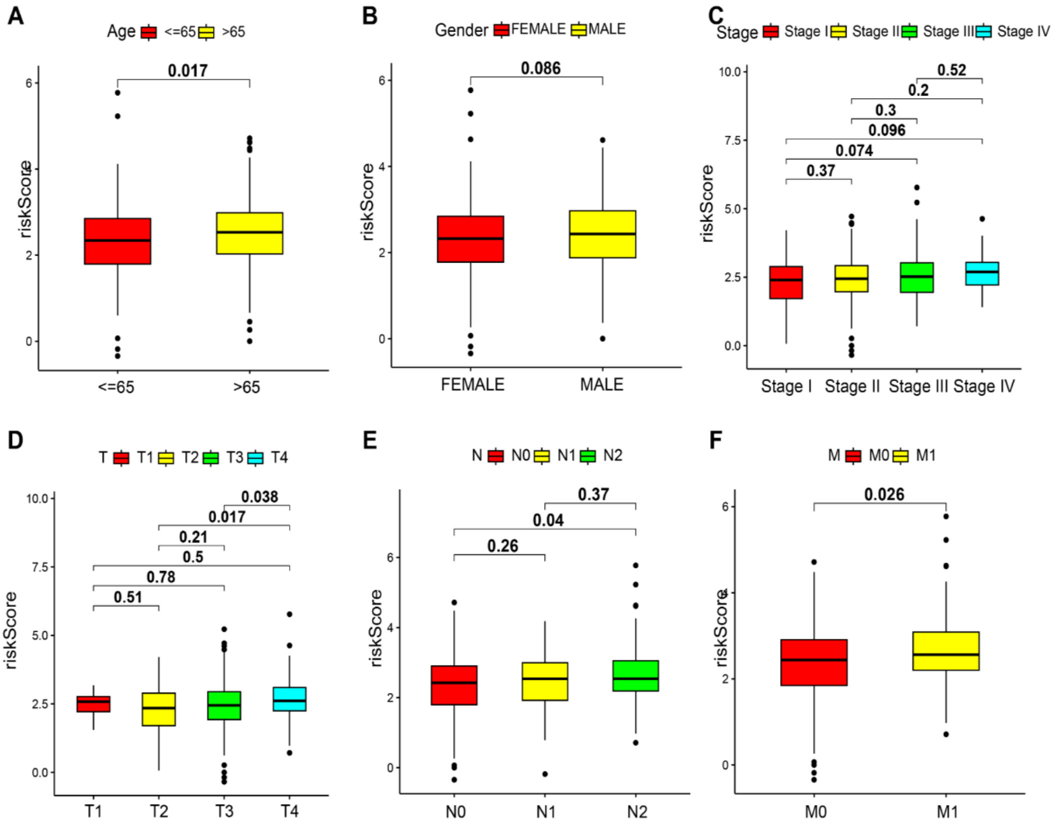

Figure S3. Correlation analysis of 29 immune-associated gene sets. Immune cells (A) and immune functions (B).

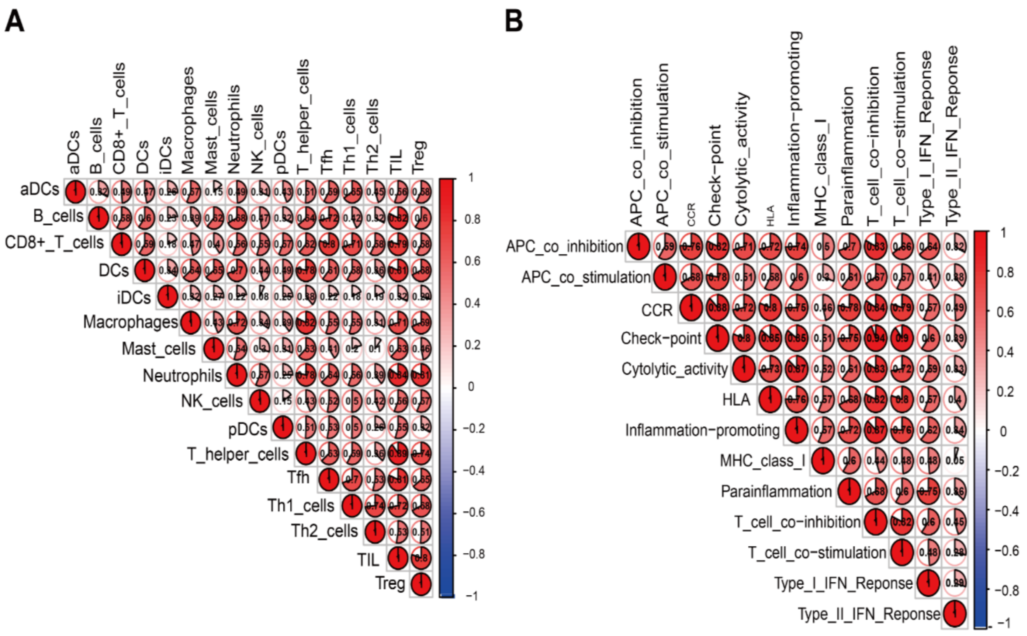

Figure S4. Analysis of tumor microenvironment of CC patients based on METTL1 and WDR4 expression. (A) Correlation analysis of METTL1 and WDR4 expression with immune cells. Comparison of immune infiltration levels of immune cells between the high- and low-METTL1 (B) and WDR4 (C) expression groups. *, p < 0.05; **, p < 0.01; ***, p < 0.001; ns, not significant.

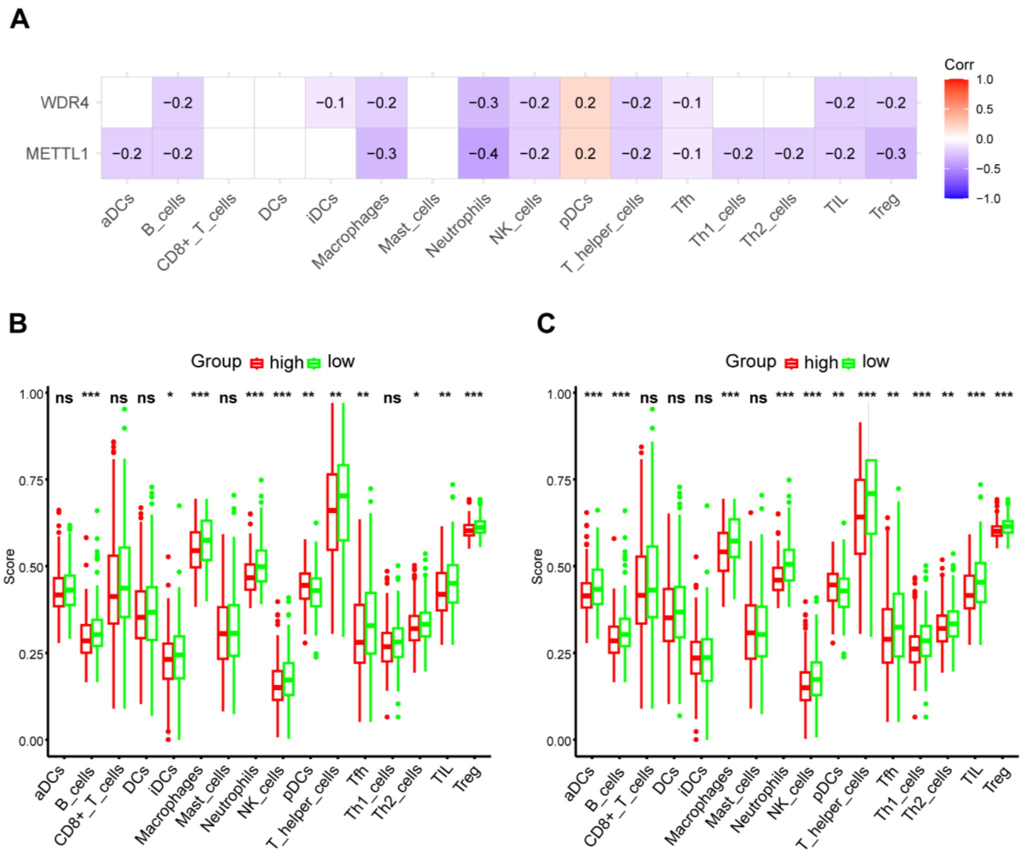

Table S1. Prognostic m7G-related miRNAs and their risk coefficients used for the construction of model.

**Prognostic miRNAs**	**Coefficient**
hsa-miR-136-5p	0.14858678
hsa-miR-6887-3p	-0.8152168
hsa-miR-195-5p	0.13534302
hsa-miR-149-3p	0.80844283
hsa-miR-4433a-5p	0.60972547
hsa-miR-31-5p	0.07243283
hsa-miR-129-2-3p	0.1965502
